# Acyl-homoserine lactone-based quorum sensing in the *Roseobacter* clade: complex cell-to-cell communication controls multiple physiologies

**DOI:** 10.3389/fmicb.2013.00336

**Published:** 2013-11-12

**Authors:** W. Nathan Cude, Alison Buchan

**Affiliations:** Department of Microbiology, University of TennesseeKnoxville, TN, USA

**Keywords:** quorum sensing, *Roseobacter*, marine bacteria, biogeochemical cycles, acyl-homoserine lactones

## Abstract

Bacteria have been widely reported to use quorum sensing (QS) systems, which employ small diffusible metabolites to coordinate gene expression in a population density dependent manner. In Proteobacteria, the most commonly described QS signaling molecules are *N*-acyl-homoserine lactones (AHLs). Recent studies suggest that members of the abundant marine *Roseobacter* lineage possess AHL-based QS systems and are environmentally relevant models for relating QS to ecological success. As reviewed here, these studies suggest that the roles of QS in roseobacters are varied and complex. An analysis of the 43 publically available *Roseobacter* genomes shows conservation of QS protein sequences and overall gene topologies, providing support for the hypothesis that QS is a conserved and widespread trait in the clade.

## Introduction

When acting as coordinated communities, bacterial populations are able to influence their local environment in manners that are unachievable by individual cells. It has been widely reported that phylogentically diverse bacteria use genetic regulatory systems, known as quorum sensing (QS) systems, to coordinate gene expression in a population density dependent manner (e.g., Fuqua et al., 2001; Pappas et al., 2004; Case et al., 2008; Ng and Bassler, 2009). Among other things, QS is hypothesized to facilitate maximal access to available nutrients through the use of exoenzymes (Vetter et al., 1998; Schimel and Weintraub, 2003), the colonization of desirable niches (Nadell et al., 2008, 2009), and competitive advantages against other organisms (Folcher et al., 2001; Chin-a-Woeng et al., 2003; Barnard et al., 2007). The chemical mediators of QS are often small molecular weight diffusible molecules (Fuqua et al., 2001; Churchill and Chen, 2011). A well-characterized type of QS uses *N*-acyl-homoserine lactones (AHLs) and appears exclusive to Proteobacteria (Case et al., 2008). Canonical AHL-QS systems produce and respond to AHLs using two proteins that mediate signal production and response, LuxI and LuxR-like proteins, respectively (Nealson et al., 1970; Ruby, 1996). The genes encoding these two proteins are often located adjacent to one another on the chromosome (Fuqua et al., 1996; Churchill and Chen, 2011; Gelencsér et al., 2012). LuxI-like proteins synthesize AHLs by cyclizing *S*-adenosyl methionine into a lactone ring and the addition of an acylated carbon chain from fatty acid biosynthesis pathways (Schaefer et al., 1996). Chain length and modification at the third carbon (either -H, -OH, or -O) allow for species or group specificity (Schaefer et al., 1996; Fuqua et al., 2001). LuxR-like proteins are response regulators that mediate the expression of genes required for communal behavior in response to intracellular concentrations of cognate AHLs (Fuqua and Winans, 1994; Fuqua et al., 1996). Activated LuxR proteins often upregulate *luxI* transcription to enhance the rate of AHL synthesis, increasing AHL concentrations, and also modulate the expression of other genes (Fuqua et al., 1996, 2001; Case et al., 2008).

AHL-based QS is common in Proteobacteria, which are abundant in coastal marine systems (Dang and Lovell, 2002; Waters and Bassler, 2005; Ng and Bassler, 2009). One of the most abundant and biogeochemically active groups of marine a α-proteobacteria is the *Roseobacter* clade (Gonzalez and Moran, 1997; Buchan et al., 2005). Roseobacters can comprise up to 30% of the total 16S rRNA genes in coastal environments and up to 15% in the open ocean (Buchan et al., 2005; Wagner-Dobler and Bibel, 2006). In coastal salt marshes, roseobacters are the primary colonizers of surfaces and mediate a wide range of biogeochemically relevant processes, including mineralization of plant-derived compounds and transformations of reduced inorganic and organic sulfur compounds (Gonzalez and Moran, 1997; Dang and Lovell, 2000; Buchan et al., 2005; Dang et al., 2008). Here, we describe some of the most compelling recent research that focuses on QS in the *Roseobacter* clade, provide a genomic perspective of QS systems in roseobacters, and highlight areas for further investigation.

## Roseobacters and quorum sensing

QS was first reported in roseobacters associated with marine snow and hypothesized to contribute to the ability of group members to colonize particulate matter in the ocean (Gram et al., 2002). Subsequent studies further demonstrated that roseobacters are prolific colonizers of a variety of marine surfaces, both inert and living, and the contribution of QS to this ability and other physiologies is of growing interest (Dang and Lovell, 2002; Berger et al., 2011; Zan et al., 2012). Characterized *Roseobacter* isolates produce diverse AHL structures with acyl chains ranging from eight to eighteen carbons in length that display varying degrees of saturation as well as all three possible oxidation states (-H, -OH, or -O) at the third carbon (for structures see Gram et al., 2002; Wagner-Dobler et al., 2005; Cicirelli et al., 2008; Mohamed et al., 2008; Thiel et al., 2009; Berger et al., 2011; Zan et al., 2012). The production of AHLs has been detected by LuxR-LacZ fusion bioreporters and mass spectrometry for several isolates (Gram et al., 2002; Wagner-Dobler et al., 2005; Martens et al., 2007; Thiel et al., 2009; Berger et al., 2011; Zan et al., 2012). Of the 43 publicly available *Roseobacter* genomes, only five lack annotated *luxI* homologs: *Oceanicola batsensis* HTCC2597, *Oceanicola* sp. S124, *Pelagibaca bermudensis* HTCC2601, Rhodobacterales bacterium HTCC2255, and *Ruegeria* sp. TM1040. All except HTCC2255, however, have *luxR* homologs (Table A2). Thus far, experimental studies of QS have primarily focused on isolated representatives of the *Ruegeria*-*Phaeobacter* branch of the *Roseobacter* clade, with the exception of the description of a diunsaturated long chain AHL produced by *Jannaschia helgolandensis* (Thiel et al., 2009), a survey of 31 AHL producing isolates (Wagner-Dobler et al., 2005), and a recent analysis of QS in *Dinoroseobacter shibae*, where QS was shown to control motility, expression of a type IV secretion system, and whether the cells divided by binary fission or budding (Patzelt et al., 2013).

Culture-based studies of bacterial symbionts of marine sponges suggest that roseobacters are the primary producers of AHLs in these systems (Taylor et al., 2004). A model for sponge-associated roseobacters has been established using *Ruegeria* sp. KLH11 (Zan et al., 2011). Studies with this strain have been informative in providing insight into the contributions of QS to host-bacterial interactions. KLH11 contains two sets of *luxRI* homologs, designated *ssaRI* (RKLH11_1559 and RKLH11_2275) and *ssbRI* (RKLH11_1933 and RKLH11_260), and a recently discovered orphan *luxI*, designated *sscI*, that is not annotated in the publically available KLH11 genome. While orphan *luxI* have not been widely described in the literature, they are best described as *luxI* homologs that are not immediately adjacent to a corresponding *luxR* homolog on the chromosome. It has been proposed that *sscI* is a recent duplication of *ssbI* (Zan et al., 2012). Heterologous expression of SsaI, SsbI, and SscI in *Escherichia coli* showed that they predominantly produce long chain saturated and unsaturated AHLs (C12-16). More specifically, SsaI produces 3O-AHL variants whereas SsbI and SscI produce 3OH-AHLs (Zan et al., 2012). The modification at the third carbon has been shown to affect the binding affinity of signaling molecules to LuxR homologs, and may allow KLH11 to finely tune its metabolism to cellular density and AHL diversity (Koch et al., 2005). KLH11 mutants deficient in QS display impaired motility, which corresponds to decreased transcription of genes encoding flagella biosynthesis machinery. The QS and motility impaired mutants form drastically thicker biofilms, suggesting when motility or QS is retarded, biofilm formation is increased (Zan et al., 2012). This may also suggest that biofilm formation may not be directly controlled by QS, but that when quorum is achieved, motility and biofilm dispersion are induced. Recent work has shown a phosphorelay system that controls motility in KLH11 is induced by QS (Zan et al., 2013). A similar phenotype has been observed in other roseobacters, and this trend may extend across the *Ruegeria*-*Phaeobacter* subgroup (Bruhn et al., 2006; Dobretsov et al., 2007).

QS-mediated physiologies have been implicated in one of the few examples of roseobacters demonstrating antagonistic behavior toward a eukaryotic host. *Nautella* (formerly *Ruegeria*) sp. R11 readily colonizes the macroalga *Delisea pulchra* resulting in bleaching and subsequent death (Case et al., 2011; Fernandes et al., 2011). To combat infection, *D. pulchra* produces halogenated furanones, which have been shown to block AHL-based QS systems in many bacterial species. Active synthesis of furanones prevents macroalgal colonization by epiphytic bacteria, including *Nautella* sp. R11. However, in the absence of halogen substrates required for furanone biosynthesis, colonization occurs rapidly (Manefield et al., 1999; Hentzer et al., 2002; Defoirdt et al., 2007). Further, it appears furanones may be effective against other potentially pathogenic *Ruegeria* spp. (Zhong et al., 2003).

QS is closely connected to antimicrobial production in several roseobacters. In *Phaeobacter* sp. strain Y4I, the regulatory controls dictating the production of the antimicrobial compound indigoidine are complex and include QS. Indigoidine production confers a competitive advantage to Y4I when grown in co-culture with *Vibrio fischeri*. Transposon insertions in either of two separate *luxRI*-like systems leads to an inability of Y4I mutants to produce wildtype levels of indigoidine and an inability to inhibit the growth of *V. fischeri*. This indicates a role for both QS systems in the synthesis of indigoidine (Cude et al., 2012). The presence of multiple QS systems in the genomes of many roseobacters suggests multi-layered control is a common feature to regulate energy intensive processes, including secondary metabolite production.

Tropodithietic acid (TDA) is a broad spectrum antimicrobial produced by multiple roseobacters in response to QS (Bruhn et al., 2005; Porsby et al., 2008; Berger et al., 2011). Genome analyses of *Phaeobacter gallaeciensis* strains isolated from geographically distant locations suggest they are capable of producing both AHLs and TDA (Thole et al., 2012). *P. gallaeciensis* 2.10 has been suggested to produce TDA in response to AHLs while colonizing the marine alga *Ulva australis*, thus protecting the alga from bacterial, fungal, and larval pathogens (Rao et al., 2007). A closely related strain, *P. gallaeciensis* DSM17395, which has also been shown to colonize *U. australis* (Thole et al., 2012), produces *N*-3-hydroxydecanoyl-homoserine lactone (3OHC10-HSL) using the LuxI homolog PgaI. 3OHC10-HSL activates the adjacent regulator, PgaR, in a concentration dependent manner, which leads to the upregulation of a TDA biosynthetic operon (Berger et al., 2011). Interestingly, in a Δ*pgaI* strain of DSM17395, addition of exogenous TDA is sufficient to upregulate TDA biosynthesis machinery, suggesting that regulation of TDA biosynthesis may involve multiple signals in some strains (Berger et al., 2011). The dual role of TDA as an autoinducer and an antimicrobial has also been demonstrated in *Ruegeria* sp. TM1040, which lacks AHL-based QS (Geng and Belas, 2010). Collectively, these data show that in addition to AHLs, roseobacters use novel autoinducers. In fact, recent investigations into novel non-fatty acyl-HSLs have shown that at least one *Roseobacter*, *Ruegeria pomeroyi* DSS-3, is capable of producing *p*-coumaroyl-homoserine lactone when grown in the presence of the aromatic lignin breakdown product *p*-coumaric acid (Schaefer et al., 2008). This discovery raises the possibility that many novel signaling molecules could be produced by roseobacters in response to available local substrates, specifically plant-derived aromatics which are primary growth substrates for roseobacters (Buchan et al., 2000; Gulvik and Buchan, 2013). The production of specific signaling molecules in response to exogenously supplied substrates suggest a single signal may convey information about both population density and environmental conditions (i.e., availability of a substrate that serves as both a source of organic nutrients and a colonizable surface), which would dictate a specific set of behaviors.

## Quorum sensing gene homology and topology

**Figure 1:**
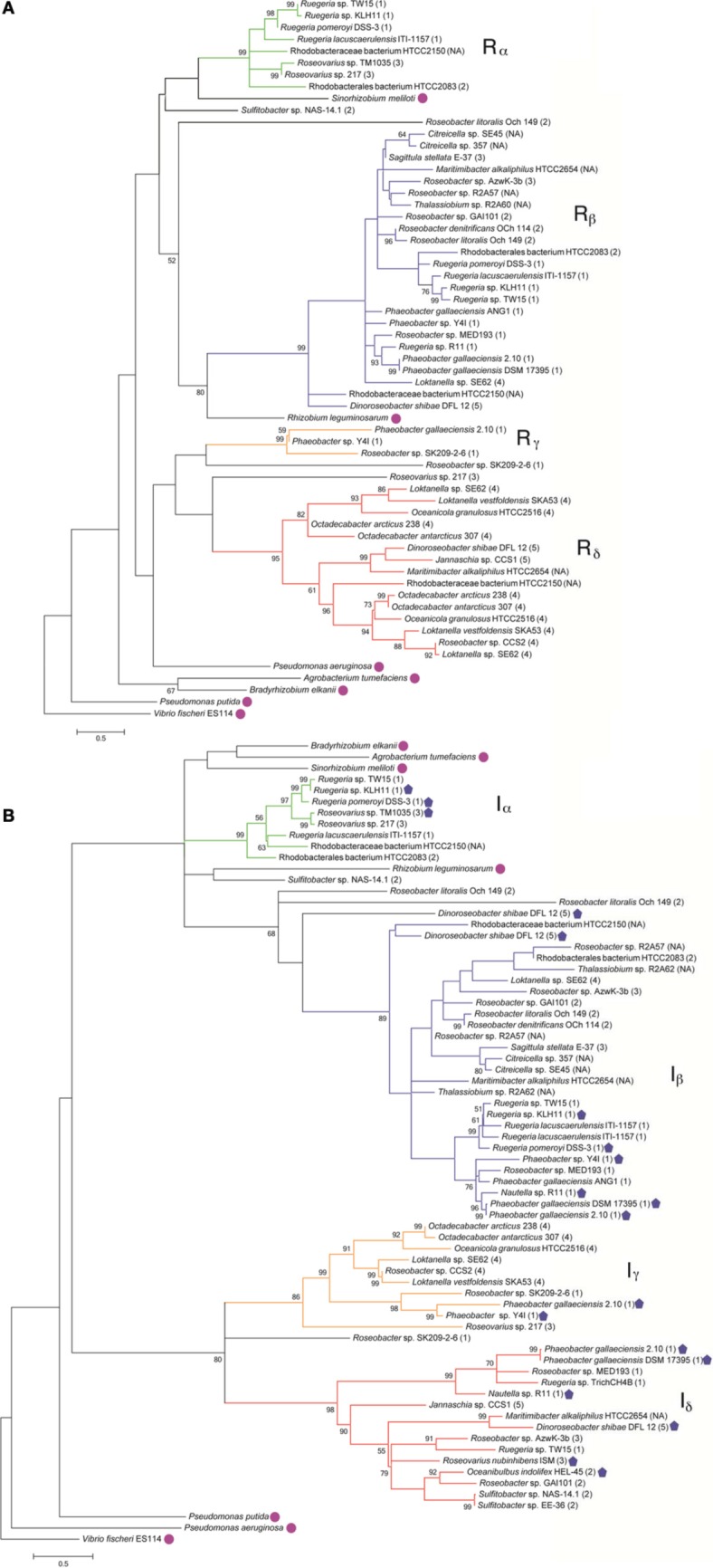
**Maximum likelihood phylogenetic trees of *Roseobacter* LuxR- (A) and LuxI-like (B) deduced amino acid sequences (see Appendix for details).** Strain designations are shown and gene locus tags of the corresponding gene sequences can be found in Table A1. The scale bar represents the substitutions per sequence position. The *Roseobacter* clade number is represented in parentheses after the organism name and follows the classification system identified in Newton et al., 2010. Proposed designations of LuxR and LuxI subgroups in roseobacters are indicated by Greek character subscript and color. Bootstrap values <50% (from 1000 iterations) are shown at branch nodes. Sequences designated with a closed pentagon indicate organisms that have been shown experimentally, by either bioreporters or mass spectrometry, to produce AHLs (Wagner-Dobler et al., 2005; Rao et al., 2006; Bruhn et al., 2007; Berger et al., 2011; Case et al., 2011; Zan et al., 2012). Sequences designated with a circle are non-roseobacters.

To understand the relatedness of AHL-based QS systems in roseobacters, we performed a phylogenetic reconstruction of the LuxI- and their neighboring LuxR-like sequences in 38 *Roseobacter* genomes. As solo LuxR homologs have been found to bind a variety of ligands, including non-AHL molecules from eukaryotic organisms (Pappas et al., 2004; Subramoni and Venturi, 2009), it is difficult to infer their contribution in AHL-based QS. Thus, *luxR* genes that are not adjacent to *luxI* genes were not included in this analysis, but they are listed in Table A2. Likely a result of the close relatedness of clade members and instances of horizontal gene transfer (HGT), many of the LuxR- and LuxI-like proteins analyzed show high sequence similarity and can be grouped together (Figures 1A,B). Our phylogenetic trees suggests there are four LuxR-like (designated R_α_, R_β_, R_γ_, and R_δ_) and four LuxI-like protein types (designated I_α_, I_β_, I_γ_, and I_δ_) found in most sequenced roseobacters, though more sequence variants may be discovered as more genome sequences become available.

**Figure 2:**
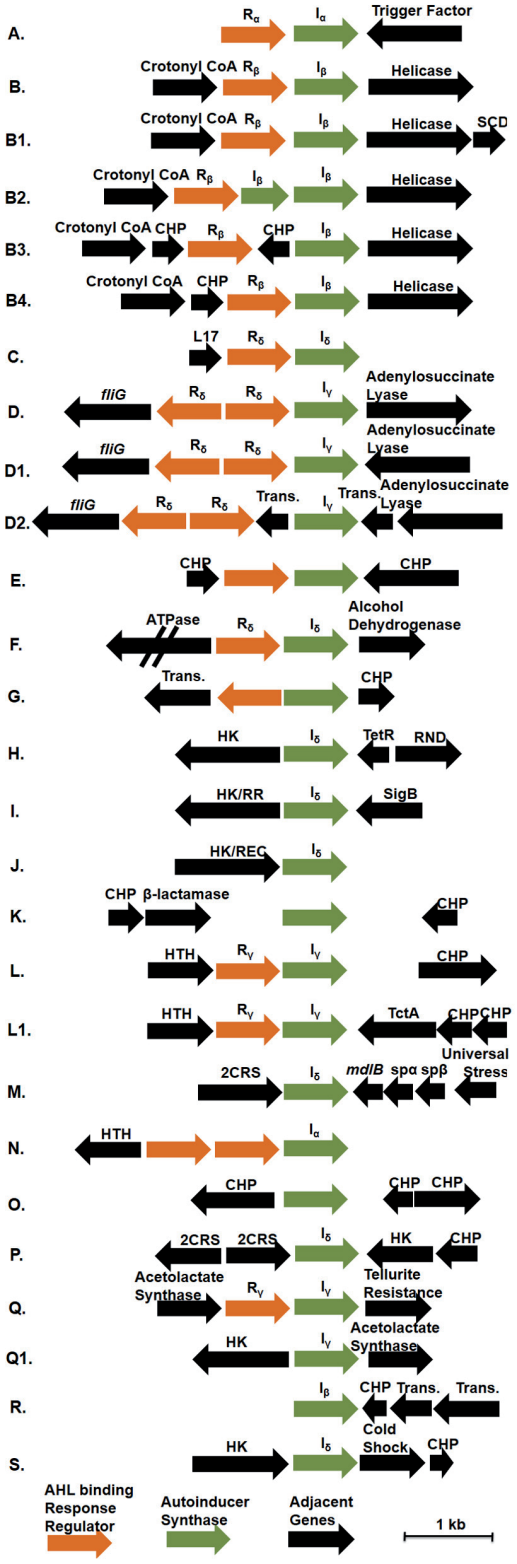
**The gene orientation of all putative *luxRI* operons in available *Roseobacter* genomes. Green arrows represent AHL synthase encoding genes (*luxI*), orange arrows represent AHL binding response regulators (*luxR*), and black arrows represent adjacent genes.** Abbreviations used: Crotonyl CoA, Crotonyl CoA reductase; HK, histidine kinase; HK/RR, hybrid histidine kinase/response regulator; HK/REC, histidine kinase with REC domain; CHP, conserved hypothetical protein; RND, RND multidrug efflux pump; Sig B, sigma B factor; SCD, short chain dehydrogenase; Trans., transposase; L17, L17 component of the 50S ribosomal protein; 2CRS, two-component regulatory system; TctA, TctA family transmembrane transporter; *mdlB*, mandelate dehydrogenase *mdlB*; spαβ, α and β subunits of sulfopyruvate decarboxylase. R_x_ and I_x_ designations above the response regulators and AHL synthases indicate their corresponding phylogentic subgroupings in Figures 1A,B, respectively. Those without R_x_ and I_x_ designations indicate unique sequences not found in the conserved groupings. The corresponding genomes that contain these topologies can be found in Table A1.

Genome analysis demonstrates that multiple conserved QS gene topologies are present within sequenced roseobacters, allowing for classification by sequence similarity and gene orientation (Figure 2 and Table A1). The most conserved gene topologies are the A and B groups, of which 28 different *Roseobacter* genomes contain one of the orientations, and three *Ruegeria* genomes contain both. Genomes that contain the A topology have highly similar LuxI and LuxR sequences (>63 and >70% similarity, respectively) and its presence in three different roseobacter subclades (defined in Newton et al., 2010) may be suggestive of HGT (Figures 1A,B). Genomes with topology A share a Trigger Factor (TF) encoding gene downstream from *luxRI* (Figure 2). The location of this TF is conserved in seven genomes. Though the function has not been examined in roseobacters, in *Vibrio cholera*, TFs play a role in the folding and secretion of proteins (Ludlam et al., 2004). The LuxI and LuxR of the A topology have been designated I_α_ and R_α_, respectively (Figure 2).

The B topology is the most prevalent among the sequenced roseobacters and is found in four variations in 24 genomes (Table A1). Like the A topology, the LuxI and LuxR protein sequences are highly similar (>73%) between the organisms that contain the B topology. This topology is found in members of all five *Roseobacter* subclades identified by Newton et al. (2010) (Figures 1A,B). The LuxI and LuxR of the B topology have been labeled I_β_ and R_β_, respectively (Figure 2). The conserved regions of the B topology include genes encoding a crotonyl-CoA reductase preceding *luxRI* and a putative ATP-dependent helicase following *luxRI*. In some organisms, crotonyl-CoA reductase interconverts unsaturated crotonyl-CoA to saturated butyryl-CoA as a precursor to fatty acid biosynthesis (Wallace et al., 1995). The helicase may be involved in DNA repair, protein degradation, or gene regulation (Snider et al., 2008). The B1 subgroup is the most abundant orientation within the B group, and contains a short-chain dehydrogenase following the helicase. This gene orientation is conserved in 14 *Roseobacter* genomes. Short-chain dehydrogenases are a large family of proteins that modify carbon chains of many substrates (Joernvall et al., 1995). The protein encoded by this gene may function to modify AHL biosynthesis substrates before or after AHL production.

Variations of the D topology are found in six *Roseobacter* genomes, all belonging to members of the *Roseobacter* subclade 4 (Figures 1A,B). These LuxI and LuxR proteins share >52 and >64% sequence similarity, respectively. The LuxI and LuxR of the D topology have been designated I_γ_ and R_δ_ (Figure 2). This topology shares two genes in common between the variations, *fliG* in the opposite orientation upstream of *luxRI* and an adenylosuccinate lyase encoding gene downstream. In *E. coli*, FliG is the flagellar motor switch that controls the spin direction of flagella (Roman et al., 1993). The characterized role of QS and motility in roseobacters was addressed previously (Zan et al., 2012), but none of the organisms containing the D topology have been investigated with respect to QS. The direct connection between QS and flagellar machinery may be an interesting avenue for future investigation. The other gene in this orientation putatively encodes an adenylosuccinate lyase, which is important in the *de novo* purine biosynthetic pathway and in controlling the levels of AMP and fumarate inside the cell (Tsai et al., 2007), suggesting purine biosynthesis may respond to QS.

The presence of orphan *luxI* genes appears common, especially in the *Sulfitobacter*, *Ruegeria*, and *Phaeobacter* genera (Table A1).The synteny of these *luxI* and their adjacent genes is conserved in the H, I, and J topologies. In organisms that have these three orientations, there is a *luxI*-like gene of the I_δ_. The LuxI of these topologies share >52% sequence similarity. Shared among the H, I, and J topologies are different types of putative histidine kinase (HK) encoding genes upstream of the orphan *luxI*, suggesting the protein is part of a two-component phosphorelay (Dutta et al., 1999; Stock et al., 2000). These genes are in the same direction as the *luxI* in H and I and in the opposite in J (Figure 2). In *Vibrio harveyi*, the hybrid two-component HK LuxN has been shown to activate gene circuits that lead to coordinated behaviors, such as bioluminescence, in response to AHLs (Freeman and Bassler, 1999; Laub and Goulian, 2007). The HKs found these topologies share modest identity with the *Vibrio harveyi* LuxN (≤26%) suggesting similar regulatory systems may be present in roseobacters. While the similarity of gene sequence does not directly predict regulatory cascades or phenotypes, the development of model systems for each of these topologies will prove valuable for comparative studies across lineage members.

## Future directions

The repertoire of chemical signals in roseobacters is anticipated to be large and result in complex chemical signaling pathways in lineage members, some of which may contribute to interspecies interactions and should be investigated further. For example, uncharacterized roseobacters have been shown to be epibionts of the abundant cyanobacterial lineage *Trichodesmium.* While AHL-based interactions between *Trichodesmium* and select epibionts have been shown to stimulate mechanisms for phosphorus acquisition in this host (Hmelo et al., 2012; Van Mooy et al., 2012), a definitive role for roseobacters in this symbiosis has not yet been demonstrated. Similarly, it has been hypothesized that QS plays a role in the switch from mutualistic to antagonistic behavior proposed for *P. gallaeciensis* in its interactions with the phytoplankter *Emiliana huxleyi* (Seyedsayamdost et al., 2011). Finally, the relationships roseobacters have with vascular plants as they colonize plant material and transform plant-derived compounds (Buchan et al., 2000; Dang and Lovell, 2000; Buchan et al., 2001) is suggestive of inter-kingdom communication, such as that found in other α-proteobacteria [e.g., *Agrobacterium tumefaciens* and *Sinorhizobium meliloti* (Hughes and Sperandio, 2008)]. Research in these areas would help elucidate the role of QS in the ability of roseobacters to colonize and interact with a diverse group of organisms.

The presence of orphan *luxR*-like genes in Proteobacterial genomes has been widely described, and their gene products have been shown to respond to AHLs and other molecules produced by other QS systems in the same organism or by other organisms (Malott et al., 2009; Patankar and González, 2009; Sabag-Daigle et al., 2012). Furthermore, it is possible that these LuxR family proteins bind structurally similar molecules that are not related to QS. In fact, it has been shown that cross-domain signaling can be mediated through LuxR homologs that bind non-AHL eukaryotic molecules (Subramoni and Venturi, 2009). In contrast, detailed studies of orphan *luxI*-like gene products are rare and are an area ripe for study. Perhaps either novel non-LuxR-like proteins or proteins encoded by genes located in distal regions of the genome (Table A2) respond to the orphan LuxI-derived AHLs. Undoubtedly, more detailed characterization of such systems will lead to a better understanding of their biological roles in roseobacters as well as other lineages.

To date, experimental studies of QS in relatively few select roseobacters have revealed complex and multi-layered control mechanisms as well as novel signaling molecules. In addition to expanding our knowledge of these characterized systems, it is our hope that future studies also broaden our understanding of currently under investigated systems within the clade and their contribution to complex multi-species interactions.

### Conflict of interest statement

The authors declare that the research was conducted in the absence of any commercial or financial relationships that could be construed as a potential conflict of interest.

## Appendix

Maximum likelihood phylogenetic trees of LuxI-like and genetically linked LuxR-like sequences from 38 published roseobacter genomes were constructed. Protein alignments of the LuxI and LuxR homologs were done using the MUSCLE algorithm with default parameters (Edgar, 2004), and manually curated. The phylogenetic trees were generated using MEGA 5.2 following published methods (Hall, 2013). The Maximum Likelihood statistical method was used with the WAG model of amino acid substitution and gamma distribution with invariant sites (G+I) selected. Gaps were handled with a 95% partial deletion data treatment, and the phylogeny was tested with 1000 bootstrap replications (Tamura et al., 2011). Bootstrap values are reported in percentages and shown at nodes where values are >50%. Groups were divided and defined by natural divisions in the trees and gene topology in the genome (Figure 2). The LuxRI protein sequences of *Vibrio fischeri* (Accession: AAQ90231.1 and AAP22376.1) were used to root the trees. LuxR and LuxI homologs of six proteobacterial species with sequence similarity to at least one roseobacter sequence in each subgroup (>30% identity) were included in the alignments to assess the validity of the groupings. The non-roseobacter LuxRI included were: *Sinorhizobium meliloti* (Accession: ABC88593.1 and CAC46417.1), *Bradyrhizobium elkanii* (Accession: WP_018273827.1 and WP_018272735), *Rhizobium leguminosarum* (Accession: YP_002281222.1 and CAD20929.1), *Agrobacterium tumefaciens* (Accession: WP_003501811.1 and AAZ50597.1), *Pseudomonas putida* (Accession: CAO85746.1 and CAO85747), *Pseudomonas aeruginosa* (CAO85753.1 and CAO85754.1.

**Table A1:** **Paired LuxRI and orphan LuxI^a^ homologs identified in the 38 sequenced roseobacters**.

**Strains**	**Gene orientation**	***luxR* gene locus**	***luxI* gene locus**
Rhodobacterales bacterium HTCC2083	A	RB2083_3272	RB2083_3255
*Ruegeria* sp. KLH11	A	RKLH11_1559	RKLH11_2275
*Roseovarius* sp. 217	A	ROS217_18272	ROS217_18267
*Roseovarius* sp. TM1035	A	RTM1035_10475	RTM1035_10485
*Ruegeria lacuscaerulensis* ITI-1157	A	SL1157_2477	SL1157_2476
*Ruegeria pomeroyi* DSS-3	A	SPO2286	SPO2287
*Ruegeria* sp. TW15	A	RTW15_010100013877	RTW15_010100013872
*Citreicella* sp. 357	B	C357_10197	C357_10192
*Citreicella* SE45	B	CSE45_4055	CSE45_4054
*Roseobacter denitrificans* OCh 114	B	RD1_1638	RD1_1639
*Sagittula stellata* E-37	B	SSE37_11169	SSE37_11164
*Ruegeria pomeroyi* DSS-3	B	SPO0371	SPO0372
*Dinoroseobacter shibae* DFL 12	B1	DSHI_2852	DSHI_2851
*Loktanella* sp. SE62	B1	LSE62_0618	LSE62_0617
*Phaeobacter gallaeciensis* 2.10	B1	PGA2_c03430	PGA2_c03440
*Phaeobacter gallaeciensis* DSM 17395	B1	PGA1_c03880	PGA1_c03890
*Phaeobacter gallaeciensis* ANG1	B1	ANG1_1316	ANG1_1315
*Phaeobacter* sp. Y4I	B1	RBY4I_1689	RBY4I_3631
*Ruegeria* sp. KLH11	B1	RKLH11_1933	RKLH11_260
Rhodobacterales bacterium HTCC2150	B1	RB2150_14426	RB2150_14421
*Roseobacter* sp. AzwK-3b	B1	RAZWK3B_04270	RAZWK3B_04275
*Roseobacter* sp. GAI101	B1	RGAI101_376	RGAI101_3395
*Roseobacter* sp. MED193	B1	MED193_10428	MED193_10423
*Ruegeria lacuscaerulensis* ITI-1157	B1	SL1157_0613	SL1157_0612
*Ruegeria* sp. R11	B1	RR11_2850	RR11_2520
*Ruegeria* sp. TW15	B1	RTW15_010100017779	RTW15_010100017784
*Roseobacter* sp. R2A57	B2	R2A57_2403	R2A57_2404
*Thalassiobium* R2A620	B2	TR2A62_3165	TR2A62_3166
			TR2A62_3167
*Maritimibacter alkaliphilus* HTCC2654	B3	RB2654_09024	RB2654_09014
Rhodobacterales bacterium HTCC2083	B4	RB2083_3265	RB2083_730
*Roseobacter litoralis* Och 149	B4	RLO149_c030690	RLO149_c030680
*Dinoroseobacter shibae* DFL 12	C	DSHI_0311	DSHI_0312
*Jannaschia* sp. CCS1	C	JANN_0619	JANN_0620
	D	SKA53_05835	SKA53_05830
*Loktanella vestfoldensis* SKA53		SKA53_05840	
*Loktanella* sp. SE62	D1	LSE62_3230	LSE62_3231
		LSE62_3229	
*Oceanicola granulosus* HTCC2516	D1	OG2516_02284	OG2516_02294
		OG2516_02289	
*Octadecabacter antarcticus* 307	D1	OA307_2044	OA307_4586
		OA307_3216	
*Roseobacter* sp. CCS2	D1	RCCS2_02083	RCCS2_02078
		RCCS2_02088	
*Octadecabacter arcticus* 238	D2	OA238_4151	OA238_2886
		OA238_3367	
*Roseobacter* sp. SK209-2-6	E	RSK20926_22079	RSK20926_22084
*Sulfitobacter* NAS-14.1	E	NAS141_01141	NAS141_01136
*Maritimibacter alkaliphilus* HTCC2654	F	RB2654_20053	RB2654_20048
*Roseovarius* sp. 217	G	ROS217_01405	ROS217_01410
*Roseobacter litoralis* Och 149	G1	RLO149_c036220	RLO149_c036210
*Sulfitobacter* NAS-14.1	H		NAS141_00695
*Sulfitobacter* sp. EE-36	H		EE36_01635
*Roseovarius nubinhibens* ISM	I		ISM_03755
*Oceanibulbus indolifex* HEL45	I		OIHEL45_00955
*Ruegeria* sp. R11	J		RR11_2017
*Roseobacter* sp. MED193	J		MED193_08053
*Ruegeria* sp. TW15	J		RTW15_010100005486
*Dinoroseobacter shibae* DFL 12	K		DSHI_4152
*Phaeobacter gallaeciensis* 2.10	L	PGA2_c18970	PGA2_c18960
*Phaeobacter* sp. Y4I	L1	RBY4I_1027	RBY4I_3464
*Phaeobacter gallaeciensis* 2.10	M		PGA2_c07460
*Phaeobacter gallaeciensis* DSM 17395	M		PGA1_c07680
Rhodobacterales bacterium HTCC2150	N	RB2150_11281	RB2150_11291
*Roseobacter litoralis* Och 149	O		RLO149_c036590
*Roseobacter* sp. AzwK-3b	P		RAZWK3B_19371
*Roseobacter* sp. SK209-2-6	Q	RSK20926_15126	RSK20926_15131
*Roseobacter* sp. GAI101	Q1		RGAI101_1101
*Ruegeria lacuscaerulensis* ITI-1157	R		SL1157_1706
*Ruegeria* sp. TrichCH4B	S		SCH4B_1938

Homologs of LuxI encoding genes were determined using BlastP to characterized proteins^b^ (E-value < e^-3^) on Roseobase (www.roseobase.org) and are consistent with the genome annotations. The LuxR gene loci listed do not represent all homologs within the genomes, but were determined based using BlastP with the autoinducer binding domain sequence from Pfam (PF03472) on Roseobase, and proximity to luxI homologs. These were also consistent with genome annotations. Gene orientations are represented in Figure 2.

a Orphan luxI homologs are defined as those that do not have an immediately adjacent luxR gene. All reported orphan luxI genes are located and at least 100 kb from the end of the draft genome contig.

^b^Vibrio fischeri LuxI (AAP22376), Agrobacterium tumefaciens TraR (AAZ50597) and Phaeobacter gallaeciensis PgaI (YP_006571842).

**Table A2:** **Putative orphan LuxR encoding genes that do not have an adjacent *luxI* on the chromosome**.

**Strains**	***luxR* gene locus**
*Citreicella* sp. 357	C357_03001
*Citreicella* sp. SE45	CSE45_1818
	CSE45_4969
*Dinoroseobacter shibae* DFL 12	Dshi_1550
	Dshi_1815
	Dshi_1819
*Jannaschia* sp. CCS1	Jann_1153
	Jann_2301
	Jann_3193
*Loktanella* sp. SE62	LSE62_3779
*Maritimibacter alkaliphilus* HTCC2654	RB2654_10983
	RB2654_03619
*Oceanibulbus indolifex* HEL-45	OIHEL45_01695
	OIHEL45_02625
	OIHEL45_13145
*Oceanicola batsensis* HTCC2597	OB2597_03302
*Oceanicola granulosus* HTCC2516	OG2516_08027
*Oceanicola* sp. S124	OS124_010100017942
	OS124_010100007975
*Octadecabacter antarcticus* 238	OA238_3367
	OA238_3623
*Octadecabacter antarcticus* 307	OA307_2044
*Pelagibaca bermudensis* HTCC2601	R2601_24964
	R2601_10664
*Phaeobacter gallaeciensis* 2.10	PGA2_c15480
	PGA2_c18970
*Phaeobacter gallaeciensis* DSM 17395	PGA1_c15590
*Phaeobacter gallaeciensis* ANG1	ANG1_869
*Phaeobacter* sp. Y4I	RBY4I_896
*Rhodobacterales bacterium* HTCC2083	RB2083_1776
*Rhodobacterales bacterium* HTCC2150	RB2150_02239
*Roseobacter denitrificans* OCh 114	RD1_3967
*Roseobacter litoralis* Och 149	RLO149_c004710
	RLO149_c036470
*Roseobacter* sp. AzwK-3b	RAZWK3B_15865
*Roseobacter* sp. CCS2	RCCS2_00422
*Roseobacter* sp. GAI101	RGAI101_670
*Roseobacter* sp. MED193	MED193_03932
*Roseobacter* sp. R2A57	R2A57_3570
*Roseobacter* sp. SK209-2-6	RSK20926_03972
	RSK20926_18892
*Roseovarius nubinhibens* ISM	ISM_09921
	ISM_15650
*Roseovarius* sp. TM1035	RTM1035_08219
*Roseovarius* sp. 217	ROS217_20327
*Ruegeria pomeroyi* DSS-3	SPO1974
*Ruegeria* sp. KLH11	RKLH11_1390
*Ruegeria* sp. R11	RR11_2316
*Ruegeria* sp. TM1040	TM1040_3102
	TM1040_1212
*Ruegeria* sp. TW15	RTW15_010100007191
*Ruegeria* sp. TrichCH4B	SCH4B_0463
	SCH4B_4179
	SCH4B_4368
	SCH4B_4682
*Ruegeria lacuscaerulensis* ITI-1157	SL1157_2844
*Sagittula stellata* E-37	SSE37_06082
*Sulfitobacter* sp. EE-36	EE36_03628
*Sulfitobacter* sp. NAS-14.1	NAS141_08556
*Thalassiobium* sp. *R2A62*	TR2A62_0664

Homologs of LuxR encoding genes were determined using BlastP with the autoinducer binding domain sequence from Pfam (PF03472) on Roseobase www.roseobase.org).
